# Concurrent Challenges in Idiopathic Hypereosinophilic Syndrome Complicating Beta-Thalassemia Major: A Case Report

**DOI:** 10.7759/cureus.56199

**Published:** 2024-03-14

**Authors:** Varun Daiya, Sunil Kumar, Sourya Acharya, Utkarsh Pradeep, Sharwari Jaiswal

**Affiliations:** 1 Medicine, Jawaharlal Nehru Medical College, Datta Meghe Institute of Higher Education & Research, Wardha, IND; 2 Dermatology, Jawaharlal Nehru Medical College, Datta Meghe Institute of Higher Education & Research, Wardha, IND

**Keywords:** management strategies, diagnostic challenges, coexistence, hematological disorders, hypereosinophilic syndrome, beta-thalassemia major

## Abstract

This case report highlights the uncommon idiopathic hypereosinophilic syndrome (HES) complicating beta-thalassemia major, presenting a diagnostic and management challenge. Beta-thalassemia major, characterized by impaired beta-globin synthesis, necessitates regular blood transfusions and iron chelation therapy. HES, a rare disorder marked by persistent eosinophilia, adds complexity to the clinical course. We present the case of a 27-year-old male with beta-thalassemia major who developed fever, weakness, and weight loss and was subsequently diagnosed with HES. Treatment involved antibiotics, blood transfusions, and corticosteroids, leading to clinical improvement. This case underscores the need to further understand the relationship between thalassemia and eosinophilia and the importance of comprehensive evaluation in patients with overlapping hematological disorders.

## Introduction

Beta-thalassemia major is a hereditary hemoglobinopathy characterized by the impaired synthesis of beta-globin chains, leading to severe anemia and a chronic need for blood transfusions [1]. This genetic disorder predominantly affects individuals of Mediterranean, Middle Eastern, and South Asian descent [1]. Management typically involves regular blood transfusions, iron chelation therapy, and supportive care to alleviate complications associated with chronic anemia [1,2]. Hypereosinophilic syndrome (HES) is a rare hematological disorder characterized by persistent eosinophilia (>1.5 × 10^9^ eosinophils/L) for at least six consecutive months, with evidence of organ involvement and excluding other known causes of eosinophilia [3]. HES can have diverse etiologies, ranging from neoplastic, autoimmune, and infectious to idiopathic origins [3,4].

The coexistence of beta-thalassemia major and idiopathic HES is an unusual clinical scenario, with limited reported cases in the literature. While thalassemia syndromes are well documented, the association of beta-thalassemia major with hypereosinophilia presents a diagnostic challenge due to the myriad of potential underlying causes for eosinophilia.

Several case reports have described the concurrence of thalassemia syndromes with eosinophilia, suggesting a possible relationship between these hematological abnormalities [5,6]. However, the mechanisms underlying this association remain unclear, necessitating further investigation. This case report contributes to the existing literature by presenting a detailed account of a patient with beta-thalassemia major who developed idiopathic HES, shedding light on the complexity of managing such overlapping conditions. Understanding the interplay between these two hematological disorders is crucial for optimizing patient care and expanding our knowledge of their potential coexistence.

## Case presentation

We present a case involving a 27-year-old male patient who sought medical attention at the outpatient department of a tertiary care hospital due to a week-long history of fever, weakness persisting for one month, and a loss of appetite also lasting one month. Upon reviewing his medical history, it was revealed that he was a previously diagnosed case of beta-thalassemia major. The initial diagnosis occurred in 2014, and since then, he has been prescribed folic acid tablets at a dose of 5 mg once a day. Additionally, the patient had a record of receiving multiple blood transfusions, with 10 units administered until 2023.

**Figure 1:**
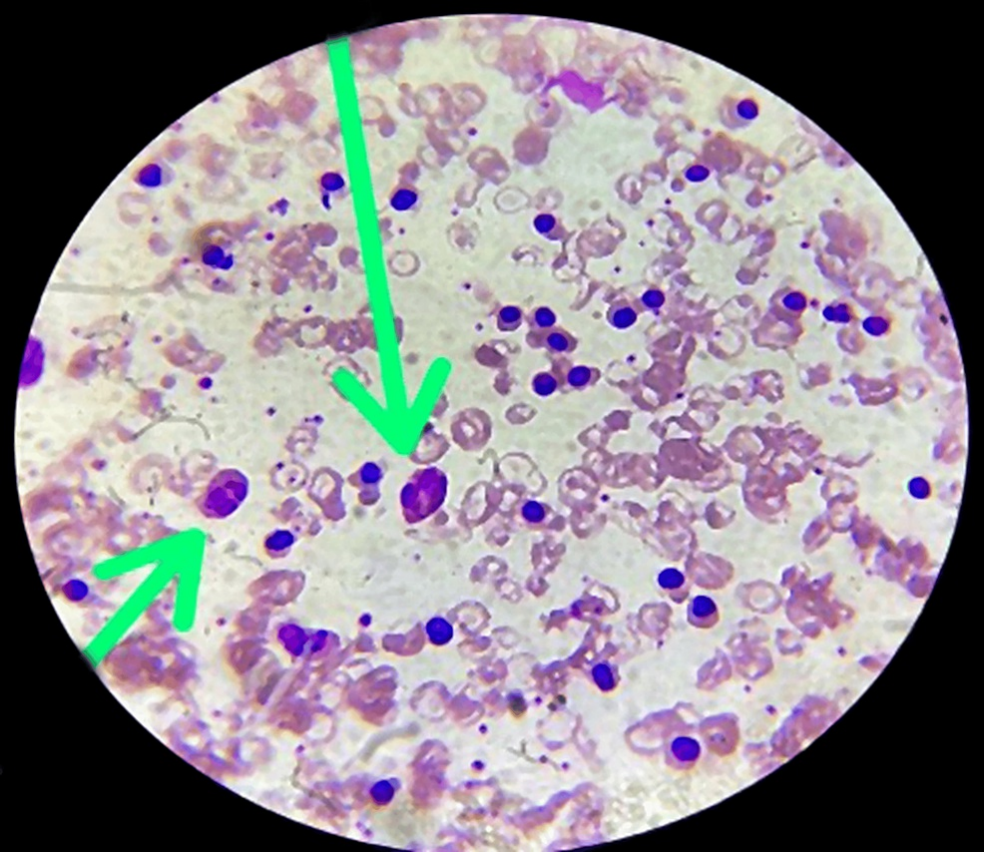
The green arrows show eosinophils observed in the peripheral smear

During the physical examination, the patient exhibited paleness and weakness, and a weight reduction of 4 kg was noted compared to the documented weight during the last follow-up visit. Consequently, the patient was recommended for further investigations, including blood tests, blood cultures, and urine examinations. A complete blood count (CBC) with a peripheral smear revealed a hemoglobin level of 6.7 gm/dL, and the peripheral smear indicated microcytic hypochromic characteristics. The total leukocyte count (TLC) was approximately 117,000 cells/cumm, and the differential leukocyte count showed 60% eosinophils (with degranulation), 27% polymorphs, 3% monocytes, 10% lymphocytes, and no basophils. Platelets were adequate on the smear, and no hemoparasites were observed. The peripheral smear strongly suggested thalassemia major with significant eosinophilia (Figure 1). Sickling tests did not reveal evidence of sickle cell anemia. Laboratory investigation is mentioned in Table 1.

**Table 1:** Laboratory investigation of the patient

Parameter	Patient’s values	Normal range
Hemoglobin	6.7 gm%	13-17 gm%
Total leukocyte count	117,000 cells/cumm	4,000-10,000 cells/cumm
Total platelet count	4.48 lakh/cumm	1.5-4.1 lakh/cumm
Mean corpuscular volume	83 fL	83-101 fL
Activated partial thromboplastin time	30 seconds	29.5 seconds
Prothrombin time	11 seconds	<20 seconds
International normalized ratio	1.05	1-1.5
Urea	29 mg/dL	19-43 mg/dL
Creatinine	0.6 mg/dL	0.66-1.25 mg/dL
Serum sodium	137 mmol/L	135-145 mmol/L
Serum potassium	4.4 mmol/L	3.5-5.1 mmol/L
Alkaline phosphatase	124 U/L	38-126 U/L
Alanine aminotransferases	25 U/L	<50 U/L
Aspartate aminotransferase	52 U/L	17-59 U/L
Albumin	4.8 g/dL	3.5-5 g/dL
Total bilirubin	5.0 mg/dL	0.2-1.3 mg/dL
Bilirubin conjugated	1.4 mg/dL	0-0.3 mg/dL
Bilirubin unconjugated	3.6 mg/dL	0-1.1 mg/dL

**Figure 2:**
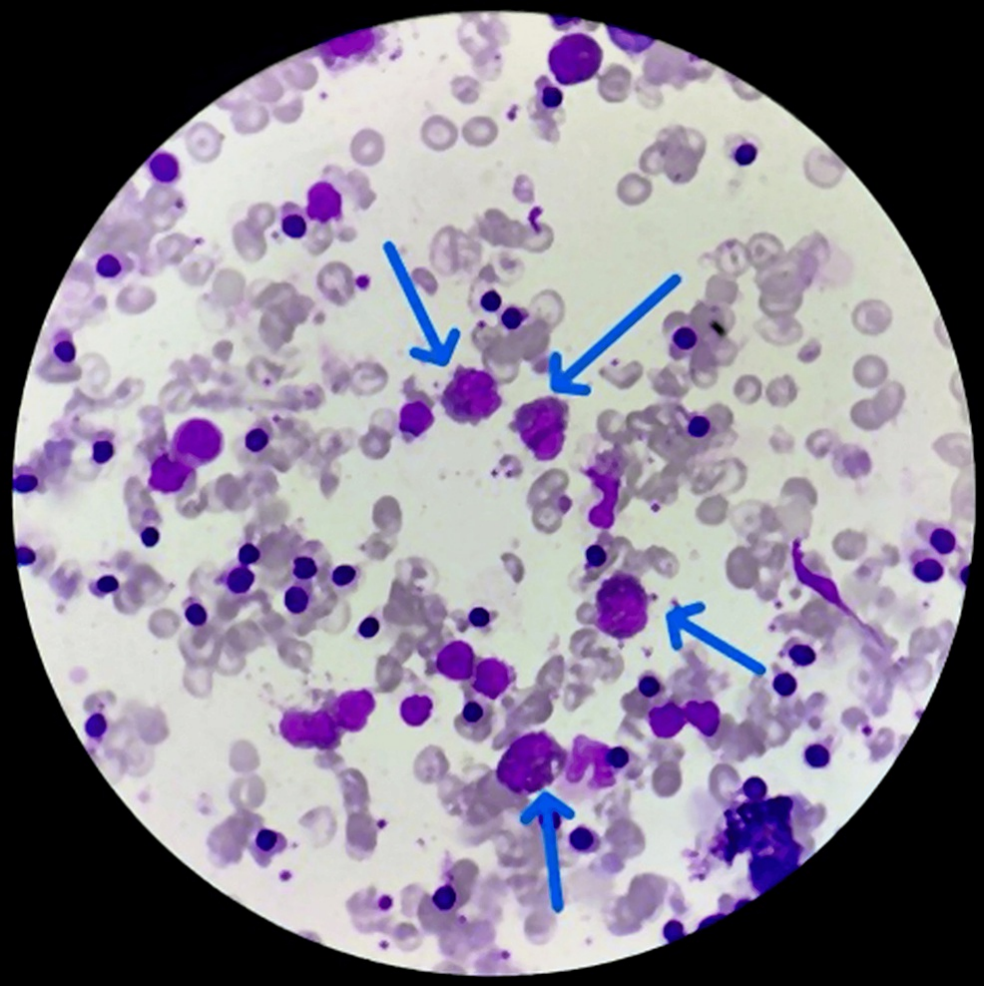
Leishman stain on bone marrow showing eosinophilia with the presence of nucleated RBC and dysmorphic anemia (blue arrows)

A blood culture was performed to investigate the cause of the fever, which showed no growth after 48 hours of incubation, and the results of a urine culture indicated insignificant growth of the organism. All these investigations yielded normal results. Consequently, we planned a bone marrow aspiration (BMA) and biopsy. The bone marrow results revealed hypercellular bone marrow with increased eosinophils in precursor form (Figure 2). Genetic analysis was not conducted because of financial constraints, including platelet-derived growth factor (PDGF) and fibroblast growth factor receptor 1 (FGFR1).

Based on the clinical and investigative findings from the peripheral smear and bone marrow, the patient was diagnosed with beta-thalassemia major with HES. He was admitted for further medical management and initiated antibiotics and antipyretics. A blood transfusion was performed following informed written consent from the patient and a relative. Post-transfusion blood samples sent for repeat CBC showed hemoglobin increased to 7.1 mg/dL. The patient was advised to remain admitted until the hemoglobin reached 8 mg/dL and the fever subsided.

Continuing the treatment regimen, the patient’s fever was reduced, and a repeat blood transfusion was conducted. The CBC report on a subsequent day indicated a hemoglobin level of 8.1 gm/dL. Given the persistent elevation of peripheral eosinophilia, systemic corticosteroid therapy in the form of tablet prednisolone 20 mg once daily was started, resulting in significant clinical improvement. The eosinophil counts became normal on a low-maintenance dose of 5 mg prednisolone on follow-up after one month. The patient was also doing well with regular follow-up.

## Discussion

The presented case of coincident idiopathic HES in a patient with beta-thalassemia major underscores the rarity of such concurrent hematological disorders and prompts further exploration into the underlying mechanisms and clinical implications of this association. Beta-thalassemia major is known for its complications arising from chronic anemia, necessitating regular blood transfusions and iron chelation therapy [7,8]. The development of eosinophilia in this patient, with 60% eosinophils observed in the peripheral smear, adds a layer of complexity to the management of thalassemia. Although a few case reports have suggested an association between eosinophilia and thalassemia syndromes [9,10], the mechanisms linking these two conditions remain speculative.

Eosinophilia is a hallmark of HES, and its occurrence in this patient raises questions about the possible interplay between thalassemia-related pathophysiology and eosinophilic disorders [11]. Previous studies have proposed a potential link between thalassemia and eosinophilia, suggesting that chronic hemolysis and inflammation associated with thalassemia may contribute to releasing eosinophils from the bone marrow [12,13]. However, comprehensive genetic analyses, including investigations into PDGF and FGFR1, were not conducted in this case, leaving the genetic basis of eosinophilia unexplored: differential diagnosis of secondary eosinophilia, clonal eosinophilia, idiopathic eosinophilia, and chronic eosinophilic leukemia [14].

The diagnostic challenges in this case highlight the importance of a thorough evaluation when encountering unexpected clinical presentations in patients with thalassemia. While the initial suspicion was for infection due to the presenting symptoms of fever and weakness, the absence of growth in blood cultures and urine tests redirected the diagnostic focus toward the hematological abnormalities. BMA and biopsy played a crucial role in confirming the diagnosis of HES, revealing a hypercellular bone marrow with enhanced premature eosinophils [14]. However, the decision not to pursue genetic analysis, including the evaluation of PDGF and FGFR1 mutations, limits our understanding of the specific molecular pathways involved in the eosinophilia observed in this case. The patient’s positive response to blood transfusions and antibiotics underscores the importance of prompt and targeted interventions in managing complex hematological conditions. Blood transfusions alleviated the anemia and reduced the eosinophil count, indicating a potential interdependence between these two hematological abnormalities [14].

## Conclusions

This case report highlights the diagnostic and management challenges posed by the rare coexistence of idiopathic HES in a patient with beta-thalassemia major. Despite the scarcely reported cases, this presentation underscores the importance of considering overlapping hematological disorders in clinical practice. The successful management of this patient through a combination of antibiotics, blood transfusions, and corticosteroids emphasizes the significance of tailored treatment strategies. Further research into this association’s underlying mechanisms and clinical implications is warranted to optimize patient care and enhance our understanding of these complex conditions.

## References

[1] Weatherall DJ, Clegg JB (1996). Thalassemia — a global public health problem. Nat Med.

[2] Cappellini MD, Cohen A, Porter J, Taher A, Viprakasit V (2014). Guidelines for the management of transfusion dependent thalassaemia (TDT) [Internet]. https://pubmed.ncbi.nlm.nih.gov/25610943/.

[3] Curtis C, Ogbogu P (2016). Hypereosinophilic syndrome. Clin Rev Allergy Immunol.

[4] Shomali W, Gotlib J (2019). World Health Organization-defined eosinophilic disorders: 2019 update on diagnosis, risk stratification, and management. Am J Hematol.

[5] Kanuru S, Sapra A (2024). Eosinophilia. StatPearls [Internet].

[6] Das JK, Gupta K, Deshmukh S, Shrivastava R (2018). A rare case of hypereosinophilic syndrome presenting with unilateral proptosis and torticollis. Indian J Ophthalmol.

[7] Weatherall DJ (2010). Thalassemia as a global health problem: recent progress toward its control in the developing countries. Ann N Y Acad Sci.

[8] Farmakis D, Porter J, Taher A, Domenica Cappellini M, Angastiniotis M, Eleftheriou A (2022). 2021 Thalassaemia International Federation Guidelines for the management of transfusion-dependent thalassemia. Hemasphere.

[9] Musharraf SG, Iqbal A, Ansari SH, Parveen S, Khan IA, Siddiqui AJ (2017). β-Thalassemia patients revealed a significant change of untargeted metabolites in comparison to healthy individuals. Sci Rep.

[10] Kuang FL (2020). Approach to patients with eosinophilia. Med Clin North Am.

[11] Patel M, Shah D, Patel S, Acharya S, Kumar S, Shukla S (2023). Hypereosinophilia syndrome: myriad presentation—a case report. J Health Allied Sci.

[12] Roufosse F, Weller PF (2010). Practical approach to the patient with hypereosinophilia. J Allergy Clin Immunol.

[13] Leru PM (2019). Eosinophilic disorders: evaluation of current classification and diagnostic criteria, proposal of a practical diagnostic algorithm. Clin Transl Allergy.

[14] Roufosse FE, Goldman M, Cogan E (2007). Hypereosinophilic syndromes. Orphanet J Rare Dis.

